# Supplementary material for: Are carbon emissions trading and green financial instruments synergistic? -Comprehensive quantitative research based on content analysis
Source: PLoS One. 2024 Mar 7;19(3):e0298601. doi: 10.1371/journal.pone.0298601 (PMC10919601; doi:10.1371/journal.pone.0298601)
Supplement: S1 Data — (ZIP) [file pone.0298601.s001.zip › Data/地级市Z_F工作报告.zip!/║╙▒▒/╨╧╠¿╩╨/└┤╫╘╣1⁄2╓┌║┼í╛┬φ┐╦╩2╛▌═°í┐_2013.docx]

各位代表：

　　我代表市人民政府向大会作工作报告，请予审议，并请各位政协委员和列席同志提出意见。

　　一、过去五年的工作回顾

　　市十三届人大一次会议以来的五年，是我市经受住各种困难风险考验、夺取改革发展新胜利的五年。在省委、省政府和中共邢台市委的正确领导下，我们坚持以邓小平理论、“三个代表”重要思想、科学发展观为指导，按照“还邢台青山绿水，走生态发展之路”的战略部署，团结进取，真抓实干，经济社会实现持续较快发展。

　　——综合实力不断增强。2012年，全市生产总值完成1532亿元、人均21361元，分别是2007年的1.7倍、1.6倍。全部财政收入170.9亿元、人均2382元，分别是2007年的2倍、1.9倍；公共财政预算收入85.6亿元、人均1194元，分别是2007年的2.7倍、2.6倍。城镇居民人均可支配收入18639元、农民人均纯收入6601元，分别是2007年的1.8倍、1.7倍。

　　——环境建设成效显著。“八大工程”深入实施，生态环境治理初见成效，百泉岩溶水位开始回升，植树造林数量实现历史性突破，全市森林覆盖率比2007年提高3.6个百分点；行政审批事项大幅削减，行政罚款“减半”，政府服务“零收费”，减轻了企业和群众负担。

　　——基础设施日臻完善。大广高速通车，邢台高铁站投入使用，邢临高速与山东实现贯通，邯黄铁路、邢汾高速、邢衡高速开工建设，京港澳高速改扩建全面铺开，公路通车总里程达1.4万公里。南水北调总干渠建设进展顺利，西线引黄工程主体竣工。

　　——城乡面貌明显改观。开展了城镇面貌三年大变样工作，建成了一批广场、游园、水系等亮点工程，七里河治理成效显著，谋划启动了邢台新区、高铁新区，城市精细化管理走在全省前列；开工建设新民居示范村396个，“垃圾围村”现象得到有效治理。

　　五年来，我们抢抓发展机遇，积极应对金融危机冲击等复杂局面，做了大量扎实有效的工作：

　　（一）增投入、上项目，夯实经济发展基础。全社会固定资产投资累计完成4770.8亿元，实施省重点项目453个，建设了中航工业重型车基地、新兴际华产业园、卡博特炭黑等66个投资超10亿元的产业项目。累计争取上级土地指标11.5万亩，通过土地置换等办法新增建设用地1.5万亩。2012年末，金融机构贷款余额突破1000亿元，新增存贷比达到95.5%；累计新增上市企业5家。

　　（二）调结构、转方式，推进产业转型升级。一是启动建设农业示范园138个，新增国家和省重点龙头企业22家，产业化经营率达到62%，2012年农林牧渔业总产值完成419.5亿元。二是规模以上工业企业数量达到894家，2012年增加值达到600.6亿元、实现利润117.9亿元；传统产业改造加快，主导产业增加值占全部工业的比重达到56%，冀中能源跻身世界500强，晶龙、旭阳跨入中国500强，市开发区光伏光热、沙河玻璃及深加工列为国家新型工业化示范基地；实施节能减排“双三十”等工程，被授予全省唯一“十一五”节能减排先进市。三是服务业增加值占全市生产总值的比重达到30.2%，2012年社会消费品零售总额完成624.1亿元；天一广场综合体、好望角物流园等项目建成投用；成立邢台银行，组建村镇银行3家，交通银行、河北银行、兴业银行在我市设立分支机构，获得河北省“金融生态市”称号。

　　（三）强功能、提品位，加大城乡建设力度。全市城镇化率达到42.8%。构建了“一城五星”大城市框架，实现了公交一体化。市区城建投资累计完成650亿元，启动改造城中村50个，完成拆迁1300万平方米，新改建道路53条，新建商住面积630万平方米，新增集中供热面积951万平方米，新增天然气用户9.7万户。县城框架进一步拉大，功能逐步完善，省级园林县城（城市）达到10个，省级卫生城达到7个。新建改造35千伏变电站219座，实现了村村通公路、村村通广播电视。

　　（四）抓园区、建平台，加快创新开放步伐。新增省级以上科技创新平台31家，高新技术企业达到35家，实施省级以上科技创新项目150个，技改投入累计1401.5亿元；新增中国驰名商标20件。完善招商平台，实现了县县有省级园区，总数达到23家，引进了中盐、中煤等一批战略投资者，五年实际利用外资14.4亿美元，进出口总额88.3亿美元，其中出口48.3亿美元。2012年，我市被授予国家科技兴贸创新基地，邢台海关开关，内陆港开港，新增市场主体2.8万个，民营经济增加值达到1026亿元。

　　（五）惠民生、促和谐，推动社会协调进步。财政累计投入662亿元改善民生，占总支出的75.3%；新增城镇就业25.7万人，转移农民就业88.7万人（次）；发放社会救助资金22.1亿元，救助困难群众291.8万人（次）；筹资6.5亿元，安置国企职工4万人；城乡居民养老保险实现制度全覆盖，职工工伤保险、失业保险实现市级统筹，城镇低保标准提高85元/月，农村低保标准提高808元/年；建设保障性住房和棚户区改造住房8.1万套，改造农村危房3.3万户；12万农村贫困人口实现稳定脱贫；解决了212万农村人口的饮水安全问题。新建、改扩建中小学857所，城乡免费义务教育全面实现，职业教育“送教下乡”办学模式在全国推广；新农合人均筹资标准由50元提高到290元，参合率达到96.8%；举办了“邢襄大舞台”惠民文化演出等活动，成功承办了省第17届大学生运动会，荣获“全国文化体制改革工作先进市”、“全国人口和计划生育综合改革示范市”称号。加强和创新社会管理，安全生产和食品药品安全形势总体平稳，人民群众安全感和满意度明显提升。双拥共建、民族宗教、外事侨务、老龄妇幼、防震减灾、应急管理、统计、档案、地方志等事业取得新成绩。

　　（六）严管理、重规范，加强政府自身建设。深入开展行政权力公开透明运行，在全省率先建立行政权力长效动态管理机制，压缩行政处罚自由裁量空间超70%，公共资源交易、电子监察等平台建成运行。尤其是2012年，开展“效能提升年”活动，市本级行政审批事项、时限分别压缩68%、75%，成为全国第一个实现政府服务“零收费”的设区市。自觉接受人大及其常委会的法律监督和工作监督、政协民主监督、社会监督和舆论监督，共办理市人大代表建议1323件、政协提案3097件，按时办复率均为100%。

　　各位代表，回顾五年历程，我们从中得到了很多有益启示：第一，解放思想是打破桎梏、实现突破的前提。每一项成绩的取得无不伴随着思想的解放，只有打开解放思想的总阀门，才能适应新形势、新要求，创出更好更快发展的新路。第二，科学发展是谋划思路、制定举措的指针。邢台的头等大事是发展，发展的核心要义是造福人民。只有以人为本，坚持一切从实际出发，按发展规律办事，既量力而行又尽力而为，才能更好更快发展。第三，改革开放是激发活力、增加动力的源泉。不改革存量不活、内力不足，不开放增量不多、外力不强。在各地竞相发展的历史潮流中，什么时候改革开放，什么时候就取得成就；而什么时候封闭保守，什么时候就留下遗憾。所以改革不能停步，开放必须加强。第四，改善环境是打牢基础、赢得竞争的根本。五年的实践表明，环境优则发展快，环境差则发展慢，甚至停滞、倒退。所以必须树立人人都是环境、人人营造环境的意识，不断改善发展环境、生态环境。第五，以民为本是开展工作、推进发展的核心。邢台人民不仅创造了灿烂辉煌的历史，也正在建设更加美好的今天。任何时候、任何发展必须始终相信群众、依靠群众、为了群众，让改革发展的成果更多惠及广大群众，获得群众支持、实现发展目的。

　　五年来，我们共同见证了邢台经济社会的快速发展、城乡面貌的巨大变化。成绩的取得，归功于省委、省政府和中共邢台市委的正确领导，得益于历届领导班子打下的良好基础，离不开市人大、市政协和社会各界的监督支持，凝聚了全市人民的智慧和汗水。在此，我代表市政府，向全市人民，向人大代表、政协委员、各民主党派、工商联、无党派人士和人民团体，向驻邢部队、武警官兵、政法干警和驻邢各单位，向关心支持邢台发展的海内外朋友，致以崇高的敬意和衷心的感谢！

　　各位代表，毋庸讳言，我们也存有一些问题。一是经济总量小，财政负担重。生产总值、财政收入等主要指标，不论是总量、人均还是增速，连年低于全省平均水平，甚至处在全省末尾；全省经济实力最弱的30个县，我市占了8个。截止2012年底，财政负债达269.4亿元，没有形成“借用还”投融资机制，债务风险加大。二是产业层次低，环境污染重。企业少、规模小；大项目少、好项目少，全市还没有一个总投资超百亿元的项目。产业链条短、内部同构化，传统产业和重化工业比重大。大气污染重，水污染和地下水超采的问题突出。三是产城布局不合理，带动能力差。中心城市规划滞后，功能不全，设施老化，交通拥堵、空气污染等“城市病”日益严重。重化工业围城，改造难度大；服务业发展不充分，支撑作用不够，集聚辐射能力弱。小城镇建管落后、吸引力小。四是社会事业欠账多，民生改善任务重。看病贵、上学难、就业压力大、食品不安全的问题客观存在，拖欠农民工工资的现象屡有发生，供热能力不足、覆盖不全，形势严峻；个别山村饮水困难。五是进取意识不强，政务环境不优。部分企业小进即满、小富即安，思想观念保守；少数干部服务意识不强，办事效率不高，法治观念淡漠，吃拿卡要现象时有发生；个别领导不思进取，缺乏担当，形式主义、官僚主义、个人主义严重。特别需要指出的是，一些政府工程由于多种原因，进展缓慢，没有达到预期目标。对这些问题，我们一定以对人民高度负责的精神，采取积极有效措施，认真加以解决。

　　二、今后五年的奋斗目标

　　今后五年，是全国深入贯彻落实党的十八大精神、全面建成小康社会的决定性阶段，时代要求、现实状况、群众期盼决定我们既要打基础，又要上水平；既要补短板，又要创优势；既要追赶，又要超车。可以说，挑战和机遇并存，困难和希望同在，我们必须坚定信心，埋头苦干，排除万难，完成任务。

　　政府工作的总体要求是：坚持以邓小平理论、“三个代表”重要思想、科学发展观为指导，全面贯彻落实党的十八大和省、市委八届三次全会精神，一手抓环境、一手抓项目，转变发展方式，加快发展速度，做大经济总量，提高经济效益，切实保障民生，促进新型工业化、信息化、城镇化、农业现代化融合发展，力争全面建成小康社会。

　　完成上述要求，中心是加强经济建设。加强经济建设，重点是狠抓项目，抓项目就是在现有基础上，由靠廉价劳动力投入、大量资源消耗和大规模投资拉动转向更多靠改革开放、人才技术、创新创造来实现高效高速增长。具体思路是：

　　坚持一大战略。即：坚定不移地实施“还邢台青山绿水，走生态发展之路”战略。

　　实现两大目标。即：力争人均生产总值、城镇居民人均可支配收入、农民人均纯收入到2016年比2010年翻一番；力争2017年经济社会发展主要指标达到全省平均水平。

　　强化三大支撑。即：装备制造、新能源、煤盐化工、新型建材、钢铁深加工、纺织服装和食品医药七大优势产业为主的产业支撑，电线电缆、羊绒及制品、轴承、汽摩配件、自行车、机械制造六大集群为主的集群支撑, 以市开发区、沙河开发区、旭阳工业聚集区三个增长极等23家省级园区为主的园区支撑。

　　破解四大瓶颈。即：破解节能减排瓶颈。提高新上项目门槛，对原有企业改造一批、转产一批、关停一批。到2017年，单位生产总值能耗下降15%，化学需氧量、氨氮、二氧化硫、氮氧化物排放量分别削减11.3%、14.5%、12.8%、16.5%。破解创新能力瓶颈。营造氛围，鼓励研发，加速技改，吸引人才，研发经费支出占生产总值的比重超过1%，新增省级以上科技创新平台21家，人才总量达到78万人，专利申请量年均增长10%以上。破解城镇化滞后瓶颈。加快区划调整，做大做强中心城市；淡化行政边界，突出经济联结；完善城镇功能，加快聚集辐射。中心城区建成区面积达到100平方公里，人口达到100万人；超20万人的中等城市达到4个；超10万人的小城市达到8个；超3万人的中心镇达到5个。破解黑龙港区域脱贫瓶颈。争取上级支持，扩大对外开放，加强教育培训，智力志气扶贫，工农一体，城乡一体，发展速度超市平均。财政收入超15亿元的县1个以上，超10亿元的县2个，其余10个县力争全部超5亿元。

　　实施五大保障。即：制定专项规划。对产业现状开展调查、摸清底数，明确发展路径和重点，打造邢台产业升级版。明确责任分工。坚持党委领导，政府负责，分别成立领导小组，结合绩效管理，目标量化到岗，责任细化到人，五年不变。倒计时间部署。按2016年、2017年两个时间节点，倒排工期，挂图作战，根据时间任务推进。定期分析研判。遵循市场规律，树立全球视野，引进智力支持，对工作进度和成效进行综合分析，及时解决发展中的问题。合力组织攻坚。四大班子上阵，企业联合实施，社会广泛参与，内外互动发展。

　　各位代表，五年时间，在历史长河中只是短短一瞬，但对于700多万邢台人民则是一次艰难的长途跋涉。我们坚信，只要“不动摇、不懈怠、不折腾”，只要方向对、路径优、不停步，只要肩并肩、手拉手、心连心，我们必能达到胜利彼岸！

　　三、2013年的重点工作和主要措施

　　今年的主要预期目标是：全市生产总值增长10%以上；全部财政收入增长12%，其中公共财政预算收入增长13%；全社会固定资产投资增长20%；规模以上工业增加值增长14%以上；服务业增加值增长10.5%，社会消费品零售总额增长15%；出口总额增长5%；实际利用外资增长12%；城镇居民人均可支配收入和农民人均纯收入均增长10%以上。围绕上述目标，以环境为核心，以项目求突破，带动活跃发展全局，重点抓好十个方面工作：

　　（一）着力改善两个环境。发展环境方面，实施简政放权，最大限度压缩行政处罚自由裁量空间，市县政府服务“零收费”和行政罚款“减半”实现全覆盖，对市直单位全面实行绩效管理，营造务实高效的政务环境；加强诚信体系建设，建立诚信档案和信用信息交换平台，弘扬激情自信、文明开放、热爱家乡、拼搏创业的邢台精神，营造真诚包容的人文环境；加强法治邢台建设，开展“法治市县”、“诚信守法企业”、“民主法治示范村（社区）”等创建活动，营造公平正义的法治环境；深入开展平安邢台建设，完善立体化治安防控体系，保持对各类违法犯罪行为的高压态势，健全利益协调、诉求表达、矛盾调处、权益保障等综合调控机制，完善社区组织功能和基础设施，确保安全生产和食品药品安全形势稳定，营造平安和谐的社会环境。生态环境方面，一是开展污染源排查。实行责任制，限期治理，对达不到排放标准的一律关停，对无证偷排的严厉打击。严格环评，杜绝“十五小”等重污染项目上马。二是深化大气污染综合整治。加大对市区大气污染企业的整治力度，将整治范围拓展到所有县级城市和工业园区，深度治理101家、搬迁入园33家、关停取缔66家，推进太阳能热利用，谋划启动热电联产项目，取代燃煤分散供暖，严格控制扬尘污染，加强机动车尾气污染治理，鼓励使用新能源汽车，逐步提高大气质量。三是扎实推进水生态系统保护与修复。实行最严格的水资源管理制度，逐步建立水生态补偿机制，实施矿山企业节水保水等10项工程，关控市区自备井100眼，力争岩溶水位继续大幅回升。四是大力开展造林绿化。实施环市区防护林带、农田林网建设等8项工程，造林绿化51.8万亩。五是继续实施农村环境卫生整治。健全农村垃圾规范化处理长效机制，引导农民改变生产生活方式，集中打造1000个高标准示范村。

　　（二）全力推进项目建设。安排市重点项目230项，年度投资550亿元，争取省重点项目数量不少于全省平均水平。一是加快重点产业项目建设。抓好93个续建项目、116个新开工项目，确保48个项目竣工投产。积极推进重大项目，新兴际华产业园完成投资10亿元，研发中心主体完工，交流中心投入使用；中航工业重型车基地完成投资7亿元，卡车生产线部分投产，军车、方舱生产线全部投产；卡博特炭黑完成投资6亿元，一期项目投产。二是加大基础设施建设力度。邯黄铁路竣工通车，邢汾高速路罗以东段建成投用，邢衡高速主体完工，环城高速基本形成，邢台机场民航复航力争开工建设，加快邢和铁路、石林高速、宁常高速等前期工作，实施邢临线等12条（段）干线公路新改建工程。三是努力破解项目难题。积极争取上级政策、土地、资金等支持，取得的人均支持数额不低于全省平均水平。严格土地管理，抓好挖潜，提高利用效率。建立银政企协调对接机制，拓宽融资渠道。四是加强项目谋划储备。围绕国家政策、产业升级、循环经济搞好谋划，项目储备力争达到500项。注重项目质量和效益，严把能评、环评关口，杜绝高耗能、高污染、低效益项目上马。

　　（三）积极扩大开放招商。一是加大招商引资力度。调动现有企业扩大投资、增上项目的积极性，让他们同样享受招商引资优惠政策；邀请邢台籍企业家回邢投资兴业，争取京先腾光刻机项目落户邢台；服务好新兴际华等央企合作伙伴，与冀中能源等省属企业合作招商，支持战略投资者自建园中园招商，力争新引进一批央企项目、产业龙头项目、战略性新兴产业项目；实施“飞地”政策，加强招商资源整合，形成全市一盘棋。二是搞好对外开放平台建设。建立与发达地区招商互动机制，加强与中原经济区融合对接，优化招商顾问团队，完善招商服务网络，组织参加好“廊坊经洽会”、“百家民企进河北”等重大活动，力争签约数量和投资额实现新突破。办好清河羊绒节、柏乡汉牡丹文化节等活动，扩大影响、吸引客商。加强海关和内陆港建设，与天津海关、港务集团加强合作，为外贸企业搞好服务。三是做好外经外贸外资工作。鼓励企业“走出去”，在国外办厂设点、兼并重组，扩大市场份额，获得所需资源。抓好外贸服务配套建设，新增自营出口企业50家，沙河玻璃、临西轴承、平乡自行车配件争取列入省级出口基地。力争全市实际利用外资4.5亿美元，引进国内市外资金448亿元。四是抓好引进项目考核和服务。加强以投资强度、环保标准、纳税数量、技术水平为主要内容的考核，提高项目引进质量。为客商提供全方位、全天候、全过程服务，促进已签约项目尽快落地。狠抓正反两方面典型，褒奖招商引资功臣，惩处损害投资环境的害群之马，营造重商、亲商、安商的氛围。

　　（四）努力壮大县域经济。一是因地制宜明确方向。在西部太行山沿线，着力发展绿色采掘业、循环制造业、优质林果业、生态旅游业，建设西部生态屏障，规范建设旭阳等生态循环工业园，开工建设南北大道，不断完善基础设施。在东部平原区，以黑龙港区域为重点，依托现有园区和产业基础，加大招商引资和项目建设力度，提升城镇要素聚集能力，壮大县城经济、园区经济、农业经济和劳务经济。实施分类指导，推进一平台县加速转型，二平台县稳步提升，三平台县跨越发展。二是壮大特色产业集群。对六大集群，加强培训辅导，加速培育龙头，帮助建立现代企业制度，引进战略投资者整合联合，横向做大、完善配套，纵向延长、加强链条，促进产业集聚、规模扩张、水平提升，销售收入年均增长20%以上。三是加快园区发展步伐。市级园区达到“七通一平”，省级园区达到“九通一平”。明确主业辅业，严格入区项目管理，新增年销售收入超百亿元园区2家，力争集群和园区列入国家和省信息化示范基地，打牢长远发展基础。

　　（五）全面提升工业经济质量和效益。一是优化产业结构。做强做大七大优势产业，提升工艺装备水平，提高产品加工深度，实施重点技改项目100项。支持新能源汽车、新材料、生物制药、节能环保等战略性新兴产业扩大规模，规模以上高新技术产业增加值增长17%。二是加大对企业的帮扶力度。为规模以上工业企业提供上下游产品对接、银企对接、要素保障等全方位服务，新增年销售收入超10亿元企业5家、规模以上工业企业150家以上。支持中小微企业发展，完善扶持政策，健全公共服务平台，新增创业辅导基地4家。深入推进全民创业，加强企业家队伍建设，支持民营企业加快发展。三是提升科技创新能力。支持中航上大等企业申报省级创新型企业，新认定高新技术企业5家，新建省级重点实验室1家、市级工程技术研究中心8家，开发省级新产品100项。重视人才的引进、培养和使用，促进政产学研结合，实施省级以上重点科技项目30项。四是扎实推进节能减排。推行清洁生产和资源再生利用，谋划建设一批生态园区。抓好新老“双三十”单位、100家重点企业的节能减排工作，单位生产总值能耗下降率和主要污染物排放削减率超额完成省定目标。

　　（六）切实加快新型城镇化进程。初步建成“借用还”城建投融资机制，力争城镇化率提高1.5个百分点。一是强化规划引领控制。科学编制我市未来5-10年城镇化发展规划，引导基础设施建设、产业布局、社会事业发展协调推进，提高城镇化发展质量；突出历史、文化、生态等邢台特色元素，打造一批精品建筑、街区和标志性景观。二是做大做强做美中心城市。一要继续建新区。邢台新区，通过招商引资实施开发，完善路网等基础设施，续建创业服务大厦；七里河新区，完善体制机制，加快建设速度；高铁新区，完善规划，封闭运行，开工建设综合客运枢纽中心。二要继续改老区。规范推进“四改”工程，力争拆迁100万平方米，加快推进回迁房建设；加强对历史文化遗存的保护，延续历史文脉。谋划建设生活垃圾焚烧处理等项目，新增天然气用户1.2万户，新增天然气供热调峰锅炉10台；对襄都路、冶金路、泉北大街雨污水管道实施改造建设，提高城市防涝能力。三要继续造环境。实施“三河五湖”、“四河截污还清”秀水工程，市区新增水面136万平方米；大力开展市区绿化，新增游园6个，建设4条休闲健身绿道，绿地面积增加46万平方米，争创国家园林城市。三是加强“一城五星”统筹。按照资源统配、设施共建、环境共创、利益共享、风险共担的思路，淡化行政区划，推进“一城五星”融合发展；统筹解决市县同城问题，力争邢台县改区实质启动；引导工业企业退市进园，谋划建设一批高效益、高附加值、高新技术项目，支持市开发区、沙河开发区、旭阳工业聚集区等加快发展，成为我市发展增长极。四是加快县城和重点镇建设。以完善设施、突出特色为重点，加快县城建设，力争新增省级园林县城4个。择优培育14个中心镇，鼓励冀中能源、德龙钢铁、旭阳焦化等大企业合并周边乡村，建设特色新型小镇，促进企业和乡镇共同发展。力争每县抓好一个商贸型、旅游型或工业型小镇。有序推进农村人口向城镇转移，积极解决失地农民就业、社保等问题，加快城乡一体化发展。五是提升精细化管理水平。市区重点抓好环境卫生、园林绿化、公共设施等8大类100项事项。推进精细化管理向县城和小城镇延伸，推广应用信息技术，加快数字化城管平台建设，以治违、治脏、治乱、治差为重点，强化对主要街道、交通环境、重点社区的综合整治。

　　（七）扎实做好“三农”工作。把“三农”工作作为全部工作的重中之重，加大投入力度，推进专业化、标准化、规模化、集约化生产经营，促进农业发展、农村繁荣、农民富裕。一是抓好农业生产。以粮食生产核心区和优质棉生产示范基地建设为重点，推广应用农业新技术，实现粮食总产90亿斤、棉花总产4.25亿斤。调整农业结构，新增市级以上畜禽养殖标准化示范场20个，创建省部级蔬菜标准园10个，发展设施蔬菜3万亩，优质干鲜果品产量达到23亿斤。二是创新生产经营体制机制。扎实推进农村产权制度改革，抓好国家、省在平乡的改革试点，开展市级试点工作，发展土地规模经营，新增土地流转面积14万亩。建立农民利益联结机制，扶持专业大户和家庭农场发展，新增市级以上农民合作示范社20个。加强技术培训，不断提高农民素质。三是提高农业产业化经营水平。扎实推进农业示范园建设，积极创建农产品品牌，力争年销售收入超5000万元龙头企业增加10家，每县至少新增1家市级龙头企业，产业化经营总量增长10%以上。四是推进扶贫开发。推广“两个组织”信贷扶贫模式，加快454个扶贫重点村整村推进工作，抓好35个扶贫开发重点项目，为1.83万户贫困家庭每户建设一项稳定增收项目，实现3万农村贫困人口稳定脱贫。五是改善农村生产生活条件。加强农业服务体系建设，抓好动植物防疫、质量检测、防灾减灾等工作。加快“两引一建”等水利工程进度，建设配套渠道及设施，新增有效灌溉面积25万亩；建设农村电网10千伏以上线路1030公里，建成1个电气化县；完成300个行政村的连村道路建设；新增省级新民居和幸福乡村建设示范点160个。

　　（八）大力提高服务业的比重和水平。一是拓展提升消费性服务业。壮大商贸服务业，支持龙头企业跨区域、多业态发展，家乐园、蓝池年营业额分别突破30亿元、50亿元，力争世贸天街、中北世纪城、红星美凯龙一期等项目建成开业，力争宁晋、沙河、隆尧、清河、内丘、威县等分别发展1家年营业额超亿元的重点商贸企业。加快发展旅游业，开工建设大元文化城，完成大峡谷项目配套、皇寺古镇改造一期、凤屏山主体工程，积极开发特色旅游商品，努力打造国家优秀旅游城市。二是加快发展生产性服务业。新组建小额贷款公司5家，引进域外金融机构、创业投资公司等进入我市，支持企业通过上市、发行债券和利用私募基金、风险投资等方式扩大社会融资，力争新增上市企业2家。支持好望角、邢业通等物流园区发展，推进冀南物流中心等项目。三是培育发展高端服务业。重点发展电子商务、创意经济、研发设计、服务外包、会展经济等。建设沙河玻璃、清河羊绒、临西轴承等7家特色产业电子商务平台，力争达到10家；启动建设桥西区服务外包产业园。四是鼓励发展家庭服务业。坚持扩规增点、提质规范，健全家政服务体系。丰富易盟家政网络服务平台内涵，支持爱晚红枫等家政服务龙头企业连锁化、标准化、规模化发展。加快邢台医专老年养护中心建设，建设社区养老中心26个、农村互助幸福院520所，支持社会力量发展养老事业。

　　（九）统筹发展社会事业。一是做好就业和社会保障工作。继续推行“72小时就业服务”，新增城镇就业5.7万人，转移农民就业6万人（次）。企业退休人员基本养老金提高10%，失业保险金提高10%，居民医保补助标准达到人均280元，逐步推进医疗保险市级统筹；城镇低保标准提高到339元/月，农村低保标准提高到1958元/年。二是发展教育事业。学前教育，坚持以政府为主，引导社会力量兴办幼儿园，新建、改扩建幼儿园219所；义务教育，强化校长、教师两支队伍建设，完成110所学校的校安工程建设任务；高中教育，突出特色、提高质量，高考本科上线率继续提升；职业教育，建立校企之间人才需求与培养的绿色通道，推进我市全国百强职业培训示范城市建设，河北机电职业技术学院新校区一期、邢台职业技术学院新校区、邢台技师学院全国高技能人才实训基地建成投用；高等教育，加强邢台学院、邢台医专等高校3个重点学科、7个特色专业和2个重点实验室建设。支持农村教育事业发展，实施“千人支教”工程，建设农村教师周转宿舍900套。采取有效措施，逐步减轻学校债务压力，让校长集中精力抓教育。三是提高卫生、计生服务水平。加快推进市人民医院、三院改扩建等项目，市二院应急楼投入使用，眼科医院迁建竣工，新增县级公立医院改革试点12个，新农合人均筹资标准提高到340元，重大疾病保障病种增加至20种。全面落实计划生育奖扶、失独家庭救助等政策，孕前优生监测实现全覆盖。四是繁荣文化体育事业。推动宁晋工笔画等文化产业快速发展，活跃书画、戏曲等文化艺术事业，邢窑、邢国墓地、唐祖陵等力争列入省或国家重点保护项目。广泛开展全民健身活动，积极创建国家高水平体育后备人才基地。推进双拥共建、民族宗教、外事侨务、老龄妇幼、防震减灾、应急管理、统计、档案、地方志等各项工作取得新进步。

　　（十）精心办好十件实事。①建学校。市区新建、改扩建小学9所、幼儿园9所，缓解小学班容量大、学前儿童入园难问题。②配村医。力争每村配备1名有资质的医务人员，建设标准化村卫生室3795个、实现村村全覆盖。③建保障房。开工建设保障性住房2.15万套、竣工1.34万套、投入使用1.2万套。④便民供应。完成“大众厨房”工程，新增放心粮油经营网点100家、万村千乡农家店100家、“一城五星”农超对接直销点100个。⑤困难救助。市区增设100个“三修”摊点和爱心摊位，解决部分弱势群体生计问题。⑥建设健身场地。改建和规范30个公共健身场点、50个社区健身示范点,新建500个村的健身场所。⑦改善农村设施。对552个帮扶村主街道实施硬化，解决25万农村人口饮水安全问题，改造农村危房1.6万户。⑧便捷出行。市区打通新华北路、兴达路南延、红星街东延、襄都路北延、永康街东延、永康街西延、莲池大街中段等7条断头路，启动实施建设大街地道桥工程，完成13条街道路灯安装，施划停车泊位500个。⑨社区服务。新增物业服务小区8个，成立市政排水应急大队，缓解无物业小区及城中村排水管网堵塞问题。⑩法律援助。对下岗职工、农民工、残疾人、未成年人等开辟绿色通道，力争随到随立案。

　　四、加强政府自身建设，改进工作作风

　　各位代表，把宏伟蓝图变为现实，实现作为“中国梦”组成部分的“邢台梦”，需要全体邢台人付出艰苦努力，更需要各级政府加强自身建设，努力改进工作作风。未来五年，我们将在以下五个方面持续努力。

　　（一）解放思想，保持干事创业激情。“破釜沉舟、以一当十”的故事就发生在邢襄大地，“激情自信”更是新邢台人精神的重要内涵。邢台最重要的资源不是煤、不是铁，而是人，人最重要的是精神，精神状态好就会群情激昂、群策群力，创出一流业绩，精神状态不好就会暮气沉沉、一盘散沙，贻误发展机遇。今天，我们的激情既来自千年文化积淀，又来自十八大的冲锋号角，既来自700多万人民的殷切期盼，又来自发展居于落后地位的强烈忧患。各级政府和公务人员必须以强烈的事业心和责任感投身工作，县与县要比，部门与部门要争，领导要冲锋在前，全体公务员要夙夜在公，人一我十、人十我百、人百我千，以勤补拙、以快补晚、以恒补慢，用激情照亮邢台的天空。

　　（二）科学决策，全面推进依法行政。首先是坚定既定发展战略不动摇，保持政策的稳定性和连续性，不另搞一套，不留半拉子工程，把政府承诺的事情，一任一任办到底。其次是坚持决策的科学化、民主化、法治化，坚决防止拍脑袋决策、照搬照抄，把上级精神、本地实际、外地经验结合起来，确保做正确的事和正确地做事，多留财富、不留包袱。第三是政府带头树立法治理念、养成法治思维、使用法治方式推动发展，主动接受人大、政协和社会监督，努力建设“法治政府”。

　　（三）转变职能，提高政府服务水平。建设服务型政府是社会主义市场经济的本质要求。当今，地区之间的发展竞争已由企业之间的竞争更多延伸为政府之间的竞争，政府服务的好与差、多与少、快与慢，成为地区竞争力的重要组成部分，政务环境成为发展的第一环境。我们一要加强学习，增强服务本领，二要不断创新，提高服务水平，更重要的是时刻保持公仆本色，强化服务意识，以服务降低邢台企业的成本，以服务提升邢台人民的尊严，以服务增强邢台综合竞争力，创造更优“软环境”。

　　（四）求真务实，确保目标落实到底。求真务实，就是一切从邢台的实际出发，不贪大求洋、好高骛远，不坐而论道、行动迟缓；就是对上级讲真话，给群众交实底儿，取得理解支持；就是对邢台人民动真情，在工作当中用真劲儿，遇到困难出实招儿，最终结果见实效；就是要用锤子钉钉子的办法抓落实，一锤一锤地打，一个钉子一个钉子地钉；就是盯住不落实的事，抓住不落实的人，没落实的事要交代清楚，不落实的人要处理到位；就是要年初建账、年中查账、年底算账，全程公开，缺一不可。

　　（五）从严执政，做到清廉清正清明。腐败践踏法治、扭曲规则、破坏公平、败坏风气、涣散精神，是邢台科学发展之大敌，必须坚决预防、遏制和清除。一是坚决简政放权，除法律另有规定的一律下放，通过减少审批和环节来减少腐败；二是阳光行政，除法律另有规定的一律公开，让权力得到监督；三是严查违法违纪案件，让腐败分子伸手必被捉，身败名亦裂；四是严格落实“中央八条”规定，艰苦奋斗，厉行节约，为人民节约每一分钱。

　　各位代表！新的征程已经开启，新的目标催人奋进。让我们紧密团结在以习近平同志为总书记的党中央周围，在省委、省政府和中共邢台市委的坚强领导下，万众一心，奋力拼搏，打造美丽邢台，建设幸福家园，为全面建成小康社会而努力奋斗！
